# Adverse Effects of Oxytocin Are More Prevalent than Those Associated with Carbetocin Administration During Cesarean Section

**DOI:** 10.3390/jcm14207211

**Published:** 2025-10-13

**Authors:** Edyta Zagrodnik, Małgorzata Szczuko, Agnieszka Kordek, Anna Surówka, Iwona Szydłowska, Beata Rzewuska, Lili Steblovnik, Maciej Ziętek

**Affiliations:** 1 Clinical Department of Anesthesiology and Intensive Care of Adults and Children, Pomeranian Medical University in Szczecin, 71-460 Szczecin, Poland; edyta.zagrodnik@pum.edu.pl; 2 Department of Bromatology and Nutritional Diagnostics, Pomeranian Medical University in Szczecin, 71-460 Szczecin, Poland; malgorzata.szczuko@pum.edu.pl; 3 Department of Neonatology, Pomeranian Medical University in Szczecin, 71-460 Szczecin, Poland; agnieszka.kordek@pum.edu.pl; 4 Department of Plastic, Endocrine and General Surgery, Pomeranian Medical University in Szczecin, 71-460 Szczecin, Poland; anna.surowka@pum.edu.pl; 5 Department of Gynecology, Endocrinology and Gynecological Oncology, Pomeranian Medical University in Szczecin, 71-460 Szczecin, Poland; iwona.szydlowska@pum.edu.pl; 6 Department of Anaesthesiology and Intensive Therapy, Pomeranian Medical University in Szczecin, 71-460 Szczecin, Poland; beata.rzewuska@pum.edu.pl; 7 Department of Perinatology, Division of Obstetrics and Gynecology, University Medical Centre, 1000 Ljubljana, Slovenia; lili.steblovnik@mf.uni-lj.si; 8 Department of General Pharmacology and Pharmacoeconomics, Pomeranian Medical University in Szczecin, 71-460 Szczecin, Poland

**Keywords:** postpartum hemorrhage, uterotonic agents, safety profile, maternal outcomes

## Abstract

**Background/Objectives**: The aim of this study is to analyze the frequency and type of subjective adverse events reported after the use of oxytocin and carbetocin in women giving birth by cesarean section. **Methods**: A total of 70 pregnant women, previously scheduled for elective cesarean section, were enrolled in this study and divided into two groups. One group (OXY) received intrapartum oxytocin at a dose of 5 IU intravenously, and the other group (CARBE) received intrapartum carbetocin at a dose of 100 μg intravenously. Both drugs were used alternately to contract the uterus immediately after the expulsion of baby during the cesarean section. **Results**: An analysis of reported subjective adverse symptoms associated with the administration of uterotonic drugs showed a higher incidence of adverse effects in the group of women receiving oxytocin compared to those receiving carbetocin. Statistical significance was observed for all of the following reported symptoms: headache, chest pain, burning sensation and heaviness in the chest, and palpitations. **Conclusions**: Although chest pain, burning and heaviness in the chest, palpitations, and headaches are more common in women giving birth by cesarean section after administration of oxytocin than after administration of carbetocin, this fact appears to be of limited clinical significance from a clinical point of view.

## 1. Introduction

Effective prevention of perinatal hemorrhage, particularly postpartum hemorrhage (PPH), is an important element of perinatal care aimed at reducing maternal morbidity and mortality rates. The identification of risk factors and the implementation of appropriate preventive procedures before the onset of symptoms are of key importance in the prevention of PPH [1]. In a study conducted by Liu et al., severe PPH was reported in 532 (1.56%) of 34,178 women, with the predominant etiology being placental pathology (55.83%). Uterine atony without concomitant retained placental tissue accounted for 38.91% of cases [2]. In turn, a systematic review and meta-analysis of 327 studies involving 847,413,451 women, conducted by Yunas et al., identified uterine atony as the most common cause of PPH (70.6%), followed by reproductive tract injuries (16.9%), retained placental tissue (16.4%), placental pathologies (3.9%), and coagulopathies (2.7%) [3]. It was also noted that 7.8% of cases had a mixed etiology. These data highlight the need to implement individualized prevention strategies targeting the most common mechanisms leading to hemorrhage, which can significantly improve perinatal safety. Caesarean section is also a well-known risk factor for bleeding and hemorrhage. Regardless of the method used to terminate a pregnancy, preventing PPH includes ensuring proper contraction of the uterine muscle. This can be achieved through the use of uterotonic drugs, among other methods. The primary method of preventing PPH is pharmacotherapy, which includes monotherapy with a single drug (oxytocin, carbetocin, methylergonovine, ergometrine, misoprostol, prostaglandin analogues, or tranexamic acid) or combination therapy, which acts in an additive manner [4]. The drug currently most commonly used to stimulate uterine contractions during labor and cesarean section is oxytocin [5]. Oxytocin circulates in the blood serum in an unbound form, and its half-life ranges from 3 to 10 min and depends on its concentration. Due to its short half-life, repeated doses are often required. A synthetic analogue of oxytocin is carbetocin [1-deamino-1-carbo-2-tyrosine(O-methyl)-oxytocin]. The drug was first described in 1987 as an analogue of oxytocin with long-lasting agonistic action. The bioavailability of the preparation after intravenous administration is 80%, and the half-life is 40 min. Uterine contraction occurs immediately after intravenous administration of the drug. Unlike oxytocin, carbetocin is administered as a single intravenous dose containing 100 µg [6,7]. An unquestionable advantage of the drug is its stronger uterine muscle contraction effect and longer duration after a single administration. The available literature contains conflicting information regarding the side effects reported by women in labor after the use of carbetocin compared to oxytocin, which has been confirmed in previously published studies [8,9,10,11,12,13]. Among the adverse effects described, it is worth noting a decrease in blood pressure, hot flashes, less frequently chest pain and shortness of breath, nausea, abdominal pain, feeling of warmth, headache, tremors, itching, metallic taste in the mouth, and back pain. In a systematic review and meta-analysis by Ai W et al. covering 17 studies involving 32,702 women, vomiting, nausea, headaches, and hot flashes were found to be the most commonly reported adverse effects of carbetocin in the treatment of PPH [14]. In contrast, in a randomized study by Mannaerts et al. conducted in 68 patients undergoing elective cesarean section, a clinically significant lower incidence of nausea was reported after administration of carbetocin compared to oxytocin, but this relationship was not statistically significant [15]. No differences were observed in blood pressure, heart rate, the need for blood pressure-raising drugs, or blood loss [15]. In turn, in the study by Stämpfli et al., which included an exploratory analysis of data on the pharmacological safety of oxytocin and its analogue carbetocin in the context of adverse hemodynamic effects, it was shown that the use of carbetocin is associated with an increased number of reports of hypertension, hypotension, and tachycardia compared to oxytocin [16]. The use of uterine muscle constricting agents during cesarean section in pregnant women is still associated with many doubts and a lack of reliable data on the safety of uterotonic drugs used during surgery. The aim of this study is to analyze the incidence of subjective adverse events reported after the use of oxytocin and carbetocin by women giving birth by cesarean section.

## 2. Materials and Methods

This study was conducted in accordance with the Declaration of Helsinki and approved by the Pomeranian Medical University Bioethics Committee, Poland (protocol code KB-0080/107/09 with date of approval of 15 June 2009). This study included 70 adult (aged 20–46) pregnant women staying at the Department of Maternal-Fetal Medicine and Gynecology, Pomeranian Medical University in Szczecin.

### 2.1. Characteristics of the Study Group

Pregnant women in singleton pregnancies between 38 + 1 and 40 + 3 weeks of gestation were eligible for this study. All women in this study had previously been scheduled for elective cesarean section for obstetric reasons (breech presentation, history of >2 cesarean sections, low-lying placenta, tokophobia, cephalopelvic disproportion, fetal macrosomia, abnormal amount of amniotic fluid) or non-obstetric indications (ophthalmic pathologies such as advanced glaucoma with large visual field defects, high myopia, conditions following eye surgery, or retinal degeneration). Pregnant women who underwent emergency cesarean section during delivery and those whose pregnancy was complicated by additional diseases that could potentially affect the analyzed parameters were excluded from this study. All cesarean sections were performed using the Misgav Ladach technique by experienced surgeons. Each pregnant woman, after receiving full information, accepted her participation in this study in writing on an informed consent form.

### 2.2. Study Design

The pregnant women were divided into two groups. The randomization of the study groups of women was carried out as follows: the group of women who underwent cesarean section on an even day of the month was designated as the OXY group (n = 34) and received 5 IU of intravenous oxytocin (Oxytocin Grindex, Joint Stock Company GRINDEX, Riga, Latvia) and the women who had a cesarean section on an odd day of the month, designated as the CARBE group (n = 36), received 100 µg of carbetocin (Pabal, Ferring Pharmaceuticals, Kiel, Germany) intravenously during the procedure. All pregnant women underwent standard laboratory tests prior to the planned procedure, such as blood group, complete blood count, coagulation system (INR, APTT) and ionogram (sodium, potassium). During the operation, immediately after delivery of the baby and cord clamping, the patients received the selected uterotonic drug. All subjective feelings and symptoms related to the administered drug reported by the women in labor were recorded.

### 2.3. Statistical Analysis

Statistical analysis was performed using Statistica PL v. 9.0 (StatSoft, Tulsa, OK, USA). Continuous variables were presented as arithmetic mean, standard deviation, median, and minimum and maximum values, while qualitative variables were presented as frequencies and corresponding percentages (fractions). The Shapiro–Wilk test was used to verify the normality of continuous variable distributions. Both randomly selected groups were preliminarily compared in terms of general characteristics using the Student’s *t*-test (arithmetic means) and Pearson’s chi^2^ test (%). The chi^2^ test was used to check whether there was a statistically significant relationship between the two qualitative variables. The significance level was set at *p* < 0.05.

## 3. Results

### 3.1. Characteristics of the Study Groups

The basic demographic and medical data of women in the OXY and CARBE groups are presented in Table 1. The analyzed data in both groups met the criteria for normal distribution of values and were homogeneous with respect to each other (Table 1). No statistically significant differences were found in the above parameters in both study groups.

### 3.2. Laboratory Parameters of Pregnant Women in the OXY and CARBE Groups

The measurements of selected laboratory parameters in the study groups are presented in Table 2. The study groups were balanced in terms of the concentration of basic ions. The results of the blood count also showed no differences between the groups.

### 3.3. Indications for Cesarean Section

The pregnancies of the women studied were terminated by cesarean section. The indications for this procedure are presented in Table 3. Both groups did not differ in terms of indications for cesarean section.

### 3.4. Course of Anesthesia and Cesarean Section

Information on the course of anesthesia and cesarean section, characterizing its most important stages in the OXY and CARBE groups, is summarized in Table 4.

Analysis of data characterizing the course of anesthesia and cesarean section did not reveal any differences between the OXY and CARBE groups. In the CARBE group, a statistical tendency toward higher intraoperative blood loss was observed, although it was not statistically significant.

### 3.5. Use of Ephedrine and Atropine During Cesarean Section

During cesarean section, 32 pregnant women required intravenous administration of atropine or fractionated doses of ephedrine. Data on the use of these drugs are presented in Table 5.

The use of ephedrine in single and repeated doses was equally common in the OXY and CARBE groups. Atropine was administered significantly more often in the OXY group, but only in the period preceding the administration of oxytocin.

### 3.6. Use of Additional Doses of Oxytocin in the OXY Group

Table 6 presents data on women in the OXY group who required additional doses of oxytocin beyond the standard dose. Each patient in the OXY group received a single intravenous dose of 5 IU of oxytocin, which was usually sufficient to contract the uterine muscle after the birth of the baby. Seven patients required an additional 5 IU of oxytocin, and two women in labor required a third dose of the drug.

### 3.7. Subjective Symptoms Reported by Women from Both Study Groups

Table 7 summarizes subjective symptoms including headaches and chest pain, burning and heaviness in the chest, and palpitations reported after administration of the uterotonic drug.

All subjective adverse symptoms listed in Table 7 were reported significantly more often by women in the OXY group who were given oxytocin.

An analysis of the reported subjective symptoms showed a significantly higher incidence of adverse effects in the group receiving oxytocin. Statistical significance was observed for all reported symptoms: headache, pain behind the breastbone, burning sensation and feeling of heaviness in the chest, and palpitations

## 4. Discussion

The use of drugs that modulate uterine contractility is associated with the risk of subjectively perceived adverse effects, as documented in numerous scientific publications.

In a study conducted by Boucher et al. [17], comparing the incidence of adverse effects after administration of oxytocin and carbetocin, the most commonly reported symptom was nausea (21.4% vs. 20.7%, respectively). Importantly, this symptom was not reported by the patients participating in this study and was not subject to further analysis. Similar results were reported in a study by Mannaerts et al. [15], which compared the incidence of nausea and vomiting in the context of a drop in blood pressure after the use of oxytocin or carbetocin. Although both drugs were shown to have a hypotensive effect with no significant differences between them, the incidence of nausea was lower in the carbetocin group (6%) than in the oxytocin group (15%), although this difference did not reach statistical significance. However, it may be clinically relevant.

In Boucher’s study, other symptoms such as retrosternal pain, pruritus, and vomiting occurred sporadically and did not reach statistical significance [17]. In contrast, in the study by Dansereau et al. [18], the incidence of chest pain after the use of oxytocin and carbetocin was significantly higher than in Boucher’s study, at 38.5% and 39.8%, respectively. In both studies, these symptoms occurred with similar frequency, with the exception of hot flashes, which appeared only in the Dansereau study [17,18].

A systematic review of the literature and meta-analysis conducted by Ai W et al. [14] identified a total of 24 different adverse effects associated with the use of carbetocin during labor. The most commonly reported symptoms were nausea and vomiting, headache, hot flashes, chills, cardiovascular symptoms, dizziness, shortness of breath, itching, metallic taste, abdominal pain, fever, chest pain, feeling of warmth, hypotension, back pain, excessive sweating, anemia, dry mouth, serious adverse events, limb pain, diarrhea, and leukocytosis (the least common).

In this study, the most commonly reported adverse reaction was a feeling of heaviness in the chest, often described by patients as subjective difficulty in breathing. This symptom occurred much more frequently than in the study by Boucher et al. [17], where it occurred only after administration of carbetocin and affected 10.3% of patients. Importantly, in the study by Dansereau et al. [18], this symptom was not reported at all, either after administration of oxytocin or carbetocin.

Another significant symptom reported by patients in our study was a throbbing, transient headache, which occurred significantly more often after the use of oxytocin (41%) than after carbetocin (19%). For comparison, in the study by Dansereau, headaches occurred in 13% and 14% of patients, respectively, while in the study by Boucher, they were reported only in the carbetocin group, in only 3.4% of patients [17,18].

Chest pain as a symptom accompanying cesarean section was also a significant problem in the study by Moran et al. [19], where as many as 42% of women experienced this type of discomfort after the use of uterotonic drugs, most of whom (82%) required opioids. Furthermore, in this group, 82% of patients had ST segment changes on ECG.

The relationship between chest pain and hemodynamic changes was also investigated by Ross et al. [20]. The authors showed that patients with ST segment depression were significantly more likely to experience tachycardia, elevated systolic blood pressure, and decreased diastolic blood pressure.

Different results were presented by Mathew et al. [21] and Żakowski et al. [22], who reported that intraoperative chest pain was rare and was not associated with changes in the ECG. However, it is worth noting that both spinal and epidural anesthesia were used in these studies, which may have made it difficult to clearly assess subjective symptoms.

In terms of methodology, our study used only spinal anesthesia, which allows for a more uniform assessment of the effect of administered uterotonic drugs on the occurrence of adverse clinical symptoms.

An analysis of available literature data indicates significant discrepancies in the incidence of adverse events associated with the use of uterotonic drugs. This suggests that the presence and severity of adverse symptoms reported by patients may be influenced not only by the pharmacological properties of the agent (oxytocin or carbetocin), but also by the type of anesthesia used. It seems likely that spinal anesthesia may be more strongly associated with retrosternal pain than epidural anesthesia, although there are currently no studies directly analyzing this aspect. In our study, all patients reporting chest pain and burning sensation also reported palpitations. However, it is worth noting that this relationship was not confirmed in the carbocysteine group, which may suggest differences in the side effect profiles of the two drugs.

In a randomized study by Moertl et al. [10], which aimed to evaluate the effect of oxytocin and carbetocin on blood pressure and heart rate, the occurrence of adverse effects was also noted. The most commonly reported symptoms were headache, vomiting, and facial flushing, the frequency of which did not differ significantly between the two study groups.

Both oxytocin and carbetocin have the ability to lower blood pressure, which should be taken into account when selecting the dose and monitoring the patient during and after administration of the drug. Optimizing the dosage is crucial in preventing adverse effects, especially in the case of oxytocin, which is often administered in repeated doses. In our study, 21% of patients required a second dose of oxytocin, and 6% required two consecutive doses.

The question of the minimum effective dose of carbetocin remains open. A study by Khan et al. [23] showed that a dose of carbetocin five times lower than the standard dose (100 µg) may be equally effective in preventing uterine atony. Given the potential relationship between dose and incidence of adverse events, further studies should focus on comparing the efficacy and tolerability of lower doses of carbetocin.

In summary, based on currently available data, it can be assumed that the incidence of adverse effects depends not only on the type of uterotonic drug used, but also on the individual reactivity of the patient and the type of anesthesia used. Further well-designed clinical trials are necessary to better understand the mechanisms influencing treatment tolerance and to optimize doses that minimize the risk of adverse effects.

However, this study has its limitations. The sample size in this study is relatively small, which significantly limits the statistical power of the analysis and thus makes it difficult to detect potential differences in the incidence of less frequently observed adverse events associated with the administered drugs. However, it should be emphasized that the purpose of recruiting this small study group was to conduct a pilot study, the main objective of which was to assess whether adverse reactions after administration of oxytocin or carbetocin are identifiable in clinical settings at all. Therefore, the results obtained should be considered preliminary and exploratory, serving as a starting point for further, larger studies with adequate statistical power. As a result, there is a risk that less common but potentially clinically significant adverse events may have remained unidentified at this stage of the analysis.

The results obtained were based solely on the subjective self-assessment of patients, without the parallel use of objective assessment methods such as electrocardiographic recording (ECG), measurements of hemodynamic parameters, or other physiological indicators. The lack of instrumental data limits the precision of the interpretation of the reported symptoms, especially in the context of potential adverse effects that could be subclinical or underestimated or overestimated by patients. This significant methodological limitation should be taken into account when analyzing and interpreting the results of this study.

The lack of validation of the reported symptoms means that their recurrence may vary slightly among patients, which affects the sensitivity and specificity of the analyzed reported symptoms.

## 5. Conclusions

Despite the limited size of the study group, we observed a significantly higher incidence of symptoms such as chest pain, burning and heaviness in the chest, palpitations, and headaches in women who received oxytocin during labor ending in cesarean section, compared to patients who received carbetocin. This suggests that oxytocin may be more frequently associated with subjectively perceived cardiovascular and systemic adverse effects, even in a small study sample. Although far-reaching causal conclusions cannot be drawn due to the small size of the analyzed population, these results indicate potential differences in the tolerance profile of the two drugs, which should be verified in further studies with greater statistical power. Based on the conflicting results of studies on this topic, it can be assumed that the incidence of adverse effects is influenced not only by the type of uterine muscle relaxant used, but also by individual sensitivity.

## Figures and Tables

**Table 1:** Medical and demographic data of pregnant women in the OXY and CARBE groups.

Parameter	Group OXY n = 34			Group CARBE n = 36			*p*-Value *
	Mean ± SD	Min.–Max.	Median	Mean ± SD	Min.–Max.	Median	
Age (years)	29.7 ± 5.3	20–39	30.0	29.3 ± 4.9	20–46	29.5	NS
Weeks of pregnancy (weeks)	38.5 ± 1.4	37–41	39.0	38.9 ± 0.9	37–40	39.0	NS
Body weight (kg)	76.5 ± 9.6	55–97	76.5	79.8 ± 12.7	57–110	78.0	NS
Height (cm)	165.3 ± 5.0	156.5–176.0	165.5	166.0 ± 4.1	158.5–182.0	164.5	NS
BMI (kg/m^2^)	28.0 ± 3.4	22.6–37.8	28.1	28.9 ± 4.1	20.1–37.0	28.3	NS

*: Student’s *t*-test, *p*-value—statistical significance, SD—standard deviation NS—not significant.

**Table 2:** Selected laboratory parameters in the OXY and CARBE groups.

Parameter	Group OXY n = 34		Group CARBE n = 36		*p*-Value *
	Mean ± SD	Median	Mean ± SD	Median	
Hemoglobin (g/dL)	12.3 ± 1.1	12.5	12.5 ± 0.9	12.5	NS
Hematocrit (%)	36.1 ± 3.1	36.1	36.4 ± 2.4	36.4	NS
Sodium (mmol/L)	137 ± 1.2	137	136.8 ± 1.2	136	NS
Potassium (mmol/L)	4.15 ± 0.3	4.1	4.16 ± 0.2	4.1	NS

*: Student’s *t*-test, *p*-value—statistical significance, SD—standard deviation NS—not significant.

**Table 3:** Indications for Cesarean Section.

Indications for Surgery	Group OXY n = 34		Group CARBE n = 36	
	n	(%)	n	(%)
Condition after >2 caesarean sections	16	(47)	15	(42)
Breech presentation	5	(15)	9	(25)
Fetal macrosomia	3	(8.5)	3	(8)
Fetal-pelvic disproportion	3	(8.5)	3	(8)
Ophthalmological indications	2	(6)	4	(11)
Tocophobia	2	(6)	1	(3)
Polyhydramnios	2	(6)	0	(0)
Oligohydramnios	1	(3)	1	(3)

**Table 4:** Course of anesthesia and cesarean section in the OXY and CARBE groups.

Variable	Group OXY n (34) avg ± SD	Group CARBE n (36) avg ± SD	*p*-Value *
Marcain Spinal Heavy dose (mg)	12.4 ± 1.5	12.8 ± 1.7	NS
Procedure time (min)	28.1 ± 7.0	29.0 ± 9.0	NS
Time to clamp the umbilical cord (min)	6.1 ± 3.0	6.2 ± 4.0	NS
Blood loss during the procedure (mL)	347.3 ± 57.0	375.5 ± 77.0	NS (0.094)
Newborn birth weight (g)	3261.2 ± 5.0	3455.6 ± 5.0	NS
Fluids administered i.v. (mL)	784.6 ± 97.6	803.6 ± 85.4	NS

*: Student’s *t*-test, *p*—statistical significance, SD—standard deviation NS—not significant.

**Table 5:** Use of ephedrine and atropine during cesarean section in the OXY (n = 34) and CARBE (n = 36) groups.

Variable		Group OXY n (%)	Group CARBE n (%)
Ephedrine 5 mg	before administering the uterotonic drug	4 (12)	4 (11)
	after administering the uterotonic drug	0 (0)	0 (0)
Ephedrine 5 mg + 5 mg	before administering the uterotonic drug	9 (26)	9 (25)
	after administering the uterotonic drug	1 (3)	2 (6)
Atropine 0.5 mg	before administering the uterotonic drug	3 (9)	0 (0)
	after administering the uterotonic drug	0 (0)	0 (0) *

*: *p* = 0.05 for the Chi^2^ test.

**Table 6:** Intravenous administration of oxytocin in the OXY group.

Oxytocin	Group OXY n = 34 n (%)
Oxytocin: standard dose 5 IU	25 (73)
Oxytocin: standard dose + 5 IU	7 (21)
Oxytocin: standard dose + 5 IU + 5 IU	2 (6)

**Table 7:** Subjective symptoms reported by women after administration of uterotonic drugs in both groups.

Subjective Symptoms	Group OXY		Group CARBE		*p*-Value *
	Symptoms Present	Symptoms Absent	Symptoms Present	Symptoms Absent	
	n (%)	n (%)	n (%)	n (%)	
Headache	14 (41)	20 (59)	7 (19)	29 (81)	0.047
Chest pain	7 (21)	27 (79)	1 (3)	35 (97)	0.019
Burning sensation in the chest	12 (35)	22 (65)	2 (6)	34 (94)	0.018
Heaviness in the chest	21 (62)	13 (38)	11 (31)	25 (69)	0.008
Heart palpitations	15 (44)	19 (56)	7 (19)	29 (81)	0.026

*: Chi^2^ Pearson test.

## Data Availability

Data are available on request from the first author.

## Abbreviations

CARBE	carbetocin
ECG	electrocardiogram
PPH	postpartum hemorrhage
OXY	oxytocin
